# Supplementary material for: Gbb Regulates Blood Cell Proliferation and Differentiation through JNK and EGFR Signaling Pathways in the Drosophila Lymph Gland
Source: Cells. 2023 Feb 19;12(4):661. doi: 10.3390/cells12040661 (PMC9954825; doi:10.3390/cells12040661)
Supplement: Supplementary file 1 [file cells-12-00661-s001.zip › cells-2159952-supplementary.pdf]

Supplementary file 1

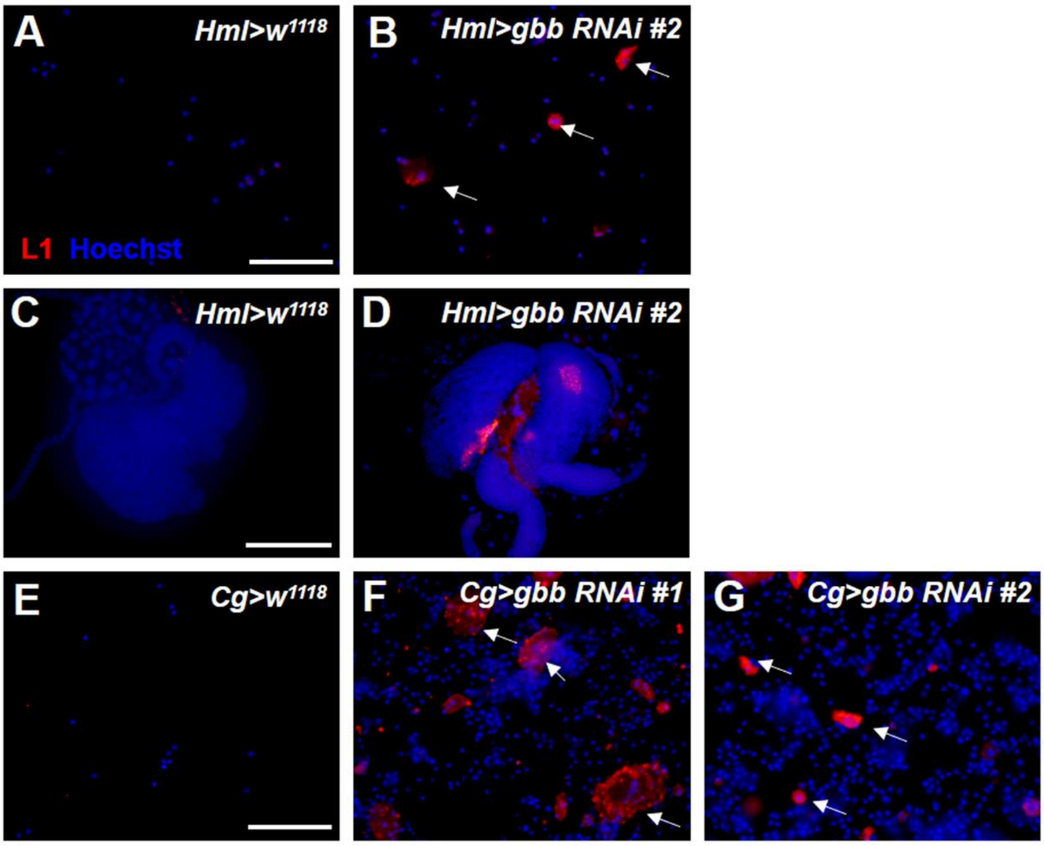

Figure S1. The knockdown of *gbb* in the maturing hemocytes can induce lamellocyte differentiation. (**A-G**) Immunostaining against lamellocyte marker L1 in the circulating hemocytes (A, B, E-G) and lymph glands (C, D) of third-instar larvae. The arrows in B, F and G indicate the lamellocyte. Scale bars: 50 µm (A, B, E-G), 100µm (C, D).

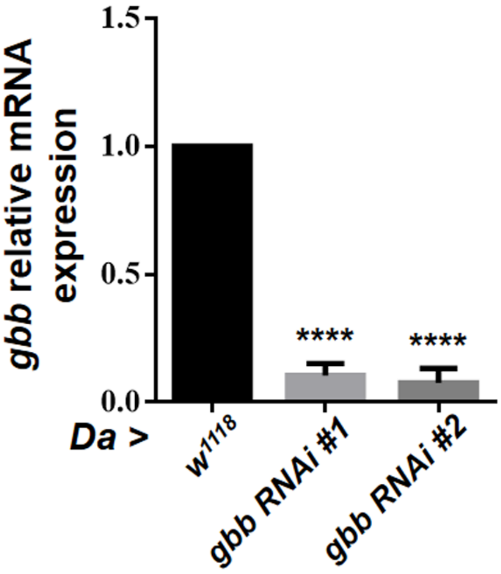

Figure S2. Real-time PCR analysis of the *gbb* level in the third-instar larvae.

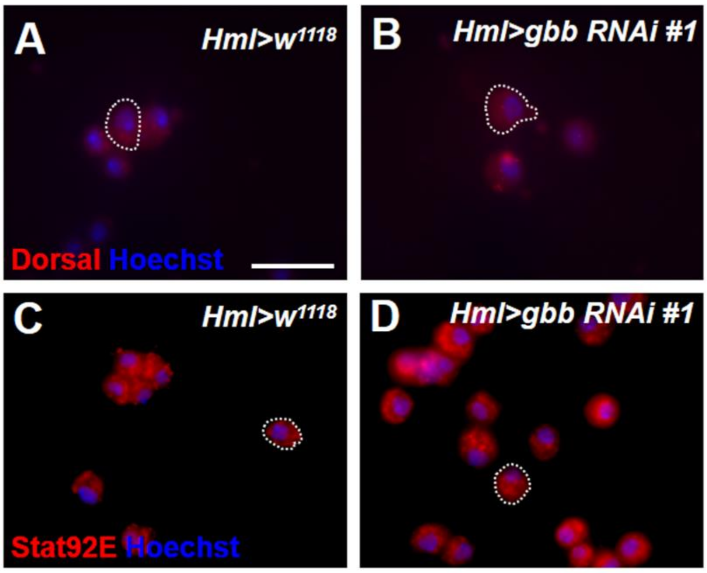

Figure S3. The knockdown of *gbb* does not induce the activation of Toll and JAK-STAT pathways. (**A, B**) Immunostaining against Dorsal in the circulating hemocytes. (**C, D**) Immunostaining against Stat92E in the circulating hemocytes. Dashed white lines in I, J, O and P outline the edges of circulating hemocytes. Scale bars: 20 μm (A-D).

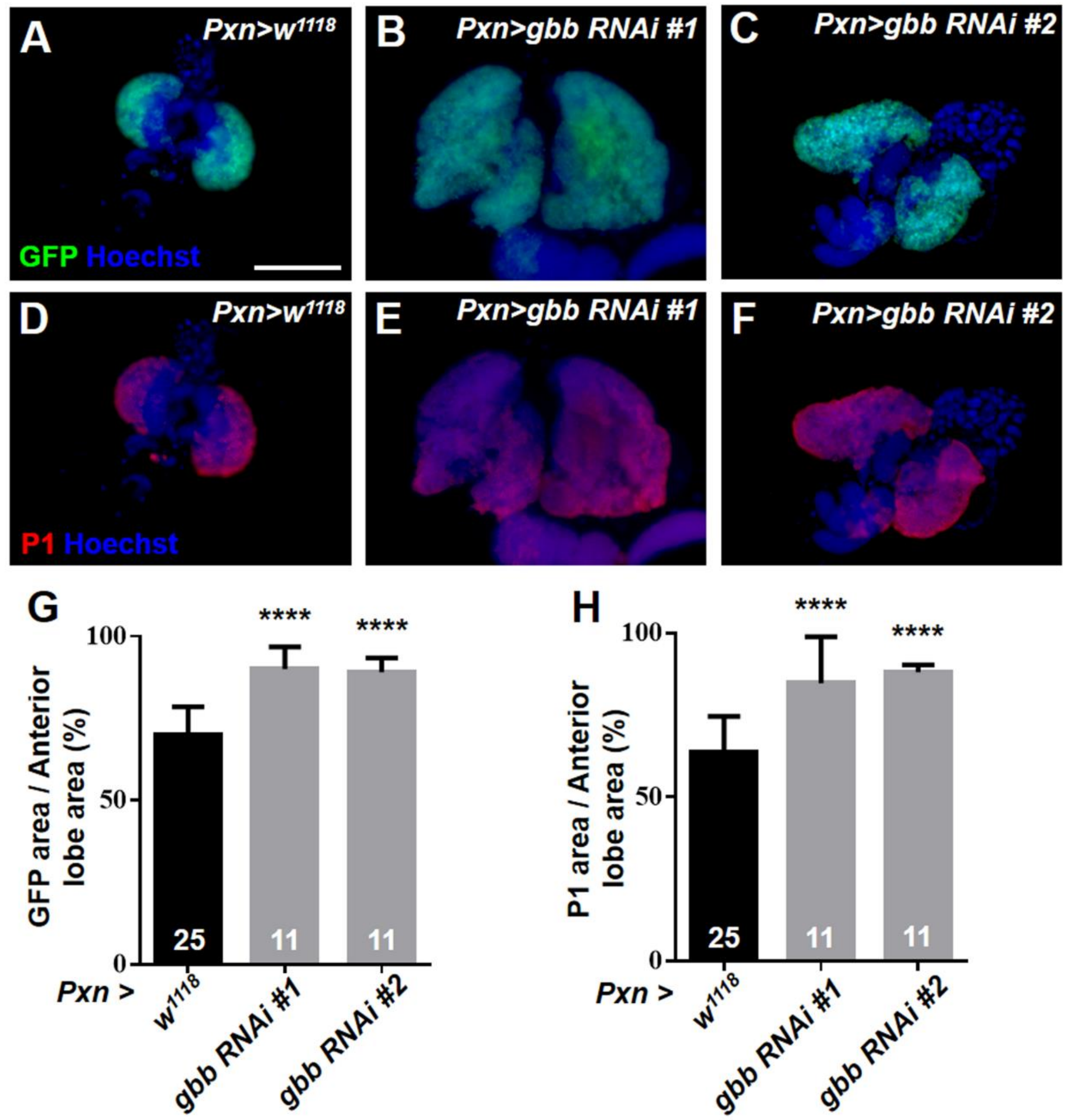

Figure S4. The knockdown of *gbb* in the CZ induces plasmatocyte differentiation. (**A-C**) The Pxn-GFP-positive area represented the area of the CZ. (**D-F**) Immunostaining for the plasmatocyte marker P1 showed that the P1-positive area was increased in *Pxn>gbb RNAi#1* (E) or *Pxn>gbb RNAi#2* lymph glands (F). (**G, H**) Quantification of the proportions of the anterior lobes occupied by the Pxn-GFP + area (G) and P1+ area (H), respectively. Scale bars: 100 μm (A-F).
